# Letter to the Editor: “Roving histological imaging for navigation-confirmed glioma negative margin by handheld endomicroscopy: a parallel controlled study”

**DOI:** 10.1097/JS9.0000000000002742

**Published:** 2025-06-23

**Authors:** Chao Wu, Pengchen Bai, Yanfei Yang, Wei Shang

**Affiliations:** a The First Affiliated Hospital of Dalian Medical University, Dalian, Liaoning, China; b Department of Neurosurgery, The First Affiliated Hospital of Dalian Medical University, Dalian, Liaoning, China

*Dear Editor*,

We read with great interest the article by Zhang *et al*^[1]^. This study innovatively applies handheld endoscopy technology (DiveScope, DS) to intraoperative resection margin assessment in glioma surgery. Through comparison with frozen section (FS) analysis, it confirms that DS demonstrates high efficiency and accuracy in real-time tissue imaging, providing a promising solution to the clinical challenge of precise boundary determination during glioma surgery. The exploration of “dynamic histopathological imaging” in the paper not only aligns with the current trend of precision tumor resection but also showcases the breakthrough application value of interdisciplinary technology in the field of neuro-oncology. Here, we share some thoughts and suggestions to further enrich the discussion. We hereby declare that this manuscript was translated and linguistic checked using artificial intelligence to ensure compliance with journal requirements. The AI use report was submitted according to the requirements of the literature “Transparency In the reporting of Artificial Intelligence-The TITAN guideline”^[2]^.

The infiltrative growth characteristics of gliomas determine a significant discrepancy between their “anatomical boundaries” and “biological boundaries” – even “negative resection margins” confirmed by imaging navigation may still harbor microscopic residual lesions. This phenomenon indicates that traditionally anatomical structure-based navigation systems urgently need deep integration with real-time assessment technologies that reflect tumor biological behavior. By virtue of its subcellular resolution imaging capability, DS achieves the first in situ observation of “cellular atypia” and “stromal microenvironment” during surgery, providing direct evidence for defining the tumor’s “biological boundary.” Future research should further integrate DS with technologies such as molecular targeted probes and metabolomic detection to construct a multimodal assessment system encompassing anatomical localization, molecular phenotype, and cellular morphology, thereby enabling more precise identification of “high-risk resection margins” with invasive potential^[3]^.

The phenomenon of “imaging-negative resection margins with residual tumor cells” discovered by DS in the study provides a unique perspective for understanding the origins of glioma recurrence after surgery. Previous research has shown that tumor recurrence often originates from single-cell invasion or micro-lesion residue at the edge of the surgical field, and these lesions are prone to being missed by conventional frozen section (FS) due to their lack of typical histological features^[4]^. The dynamic scanning capability of DS can not only be used for intraoperative decision-making but also serve as a research tool to explore whether inflammatory cells or the extracellular matrix in the microenvironment are involved in the immune escape of residual lesions. By performing single-cell sequencing or spatial transcriptomic analysis on “suspicious regions” identified by DS, it can reveal key molecular pathways underlying glioma invasion, providing a basis for screening targets for postoperative adjuvant therapy^[5]^.

This study has set an important benchmark for intraoperative margin assessment in neuro-oncology, undoubtedly representing a critical step in advancing the surgical treatment of gliomas from “anatomical resection” toward “biological precision.” We extend our sincere gratitude and appreciation to the authors for their efforts in this research and once again acknowledge the contributions of both the authors and the editorial team to the field’s progress. We look forward to their continued leadership in driving innovations in intraoperative precision diagnosis and treatment through future studies.

## Data Availability

None.
